# Higher use of fixed-bearing over mobile-bearing and posterior-stabilized over medial pivot designs in total knee arthroplasty (TKA): a systematic comparative analysis using worldwide arthroplasty registers from England and Wales, Australia, Norway, New Zealand, Germany and Switzerland

**DOI:** 10.1007/s00402-022-04410-8

**Published:** 2022-03-18

**Authors:** Ulrike Wittig, Maximilian Moshammer, Ines Vielgut, Georg Hauer, Patrick Reinbacher, Andreas Leithner, Patrick Sadoghi

**Affiliations:** Department of Orthopaedics and Trauma, Medical University of Graz, Auenbruggerplatz 5a, Graz, Austria

**Keywords:** Total knee arthroplasty, TKA, Arthroplasty register, Epidemiology

## Abstract

**Introduction:**

The aim of this study was to compare the use of mobile-bearing, fixed-bearing, posterior-stabilized (PS) and medial pivot design to describe epidemiological differences and subsequent outcomes.

**Materials and methods:**

A systematic literature search was performed using the NORE website to identify the relevant arthroplasty registers. Inclusion criteria were the following: (1) reports had to be publicly available, (2) reports had to be written in German or English language, (3) differentiation between mobile- and fixed-bearing, posterior-stabilized, and if possible, medial pivot designs had to be possible from the present reports, and (4) data had to be reported for at least three consecutive years and the latest report had to be from the year 2020 to retrieve recent data.

**Results:**

Six registries (England and Wales, Australia, Norway, New Zealand, Germany, Switzerland) offered sufficient data according to the inclusion criteria. In all countries, the dominant type of bearing used for total knee arthroplasty (TKA) was fixed-bearing, with percentages ranging from 60.8% to 84.1% in 2018, 63.6% to 85.7% in 2019 and 66.2% to 87.4% in 2020. A large variation was observed concerning mobile-bearing design, which showed a range from 2.8% to 39.2% in 2018, 2.6% to 36.4% in 2019 and 2.9% to 33.8% in 2020. Some variation was found regarding the use of PS TKA, as its percentage frequency ranged from 9.7% to 29.2% in 2018, 9.8% to 29.4% in 2019 and 10.1% to 28.5% in 2020. Medial pivot design had a share of 9.1% in 2018, 8.6% in 2019 and 8.4% in 2020 in Australia, while it only accounted for 1.4% in 2018, 2.1% in 2019 and 2.5% in 2020 in Germany.

**Conclusion:**

The comparison of arthroplasty registers from England and Wales, Australia, Norway, New Zealand, Germany and Switzerland revealed large differences regarding the application of posterior-stabilized designs, but also common ground considering the overwhelming use of fixed-bearing inserts, which, when inserted correctly, eradicate the potential complication of bearing dislocation. Arthroplasty registers offer a real-world clinical perspective with the aim to improve quality and patient safety.

## Introduction

The establishment of arthroplasty registers was a result of the development of total joint arthroplasty in the 1970s and subsequent projects for documentation purposes, which eventually developed to regional or national registries [1–4].

The main aim of arthroplasty registers is to evaluate the outcome of joint arthroplasties concerning implant-related factors, surgical technique and patient-related factors, and consequently to determine the efficacy and detect possible failures and disadvantages [5–10].

However, no systematic comparisons of different design concepts in total knee arthroplasty (TKA) have been published, so far. Nonetheless, the actual epidemiology of different design concepts with their advantages and disadvantages including mobile- or fixed-bearing, posterior-stabilized or medial pivot designs is of great interest to the optimization of patient care.

Mobile-bearing and fixed-bearing TKAs form two groups based on different fundamental design principles [11]. In fixed-bearing TKA, the polyethylene tibial insert is quite flat and locked with the tibial tray and only allows some small rotations and translations [12]. Mobile-bearing was introduced in the 1980s to allow rotation of the insert around the longitudinal axis, for which it was also given the name “rotating platform”, and anterior–posterior translation between the insert and the tibial tray, similar to the function of the menisci, for which it is also called “meniscal bearing”. Potential advantages may include reduced insert wear, less risk of loosening, fewer revisions and better clinical outcome [13–18]. After removal of the posterior cruciate ligament (PCL), a posterior-stabilized (PS) design is used. PS TKA is supposed to enable a conforming articulation between femur and tibia and to simplify ligament balancing [19–23]. However, both the cruciate-retaining (CR) and the PS design change normal knee kinematics, which leads to an abnormal anterior sliding of the femoral component on the tibial plateau called “paradoxical motion” [24]. As several studies showed, under normal circumstances, a posterior sliding of the lateral femoral condyle and a pivoting movement on the medial compartment can be observed. This anatomical understanding of a concave shape on the medial plateau and a convex shape on the lateral as well as different loads between the two compartments, where 60% of body weight is transferred through the medial side, and a knee that is more stable on the medial side than the lateral, led to the concept of medial pivot design [25, 26].

The aim of this study was to compare the use of different concepts in total knee arthroplasty including mobile-bearing, fixed-bearing, posterior-stabilized and medial pivot design to describe epidemiological differences and subsequent outcomes. Our hypothesis was that due to the new concepts of alignment theories, the use of fixed-bearing designs was higher than the use of mobile-bearing designs and that there was still higher use of posterior-stabilized than medial pivot designs due to the more recent establishment of the latter.

## Materials and methods

### Search strategy

The NORE website for European Arthroplasty Registers was screened to identify the existing worldwide registers included in our study [27]. In addition, a free-hand search using the search keywords “(arthroplasty register) OR (knee arthroplasty register)” was performed via Google. The final search date and final date, when all registries were accessed, was December 20, 2021. This method has been described in previous studies [2, 3].

Arthroplasty registers had to fulfill the following inclusion criteria to be considered for evaluation: (1) reports had to be publicly available, (2) reports had to be written in German or English language, (3) differentiation between mobile- and fixed-bearing, posterior-stabilized and if possible medial pivot designs had to be possible from the present reports, and (4) data had to be reported consistently for at least three consecutive years and the latest report had to be from the year 2020 to retrieve recent data. Exclusion criteria were annual reports containing incomplete data between 2018 and 2020 and reports not available in German or English language.

Initially, 27 national or regional knee arthroplasty registers were identified.

### Study selection and outcomes

The relevant arthroplasty registers that fulfilled the inclusion criteria were searched to find the annual report from 2020 as well as the reports from the two preceding years. Data were extracted with respect to the number of TKA procedures performed, share of mobile- or fixed-bearing design and proportions of posterior-stabilized or medial pivot designs. The number of TKA implantations was normalized to the number of inhabitants of the respective country. Absolute and relative numbers of mobile- and fixed-bearing concepts, posterior-stabilized and medial pivot designs were extracted and absolute data were then normalized to the number of total TKA implantations of the respective country. These parameters were analyzed for each arthroplasty register in duplicate and compared against one another. Disagreement was resolved by discussion or, if necessary, by the decision of the senior author according to the PRISMA guidelines [28].

All arthroplasty registers that satisfied the inclusion criteria presented their data in the form of an annual report for each year separately.

However, if registers only gave information about the overall brand name that was used, where the implant could have been applied in either ways, inclusion of the register in this study was not possible. An exception was made for the use of medial pivot design, as it was not classified separately like for mobile- or fixed-bearing and posterior-stabilized designs in some registers. Hence, differentiation according to the registry data had to be possible at least between mobile- or fixed-bearing and posterior-stabilized designs.

Finally, six (England and Wales, Australia, Norway, New Zealand, Germany, Switzerland) registers offered sufficient data.

### Data analysis

Descriptive statistics were used to analyze registry data. In this study, to compare all included registers, annual total number of TKA implantations per 100,000 inhabitants as well as the share of mobile- and fixed-bearing, posterior-stabilized and medial pivot designs was calculated. Population data on the internet were screened to find the respective number of inhabitants in the respective years in which report data were collected [29]. However, of course, this is limited by the fact that not every TKA implanted in a country is detected in the respective registry.

In general, the present review focused on descriptive analysis of the results.

## Results

**Fig. 1:**
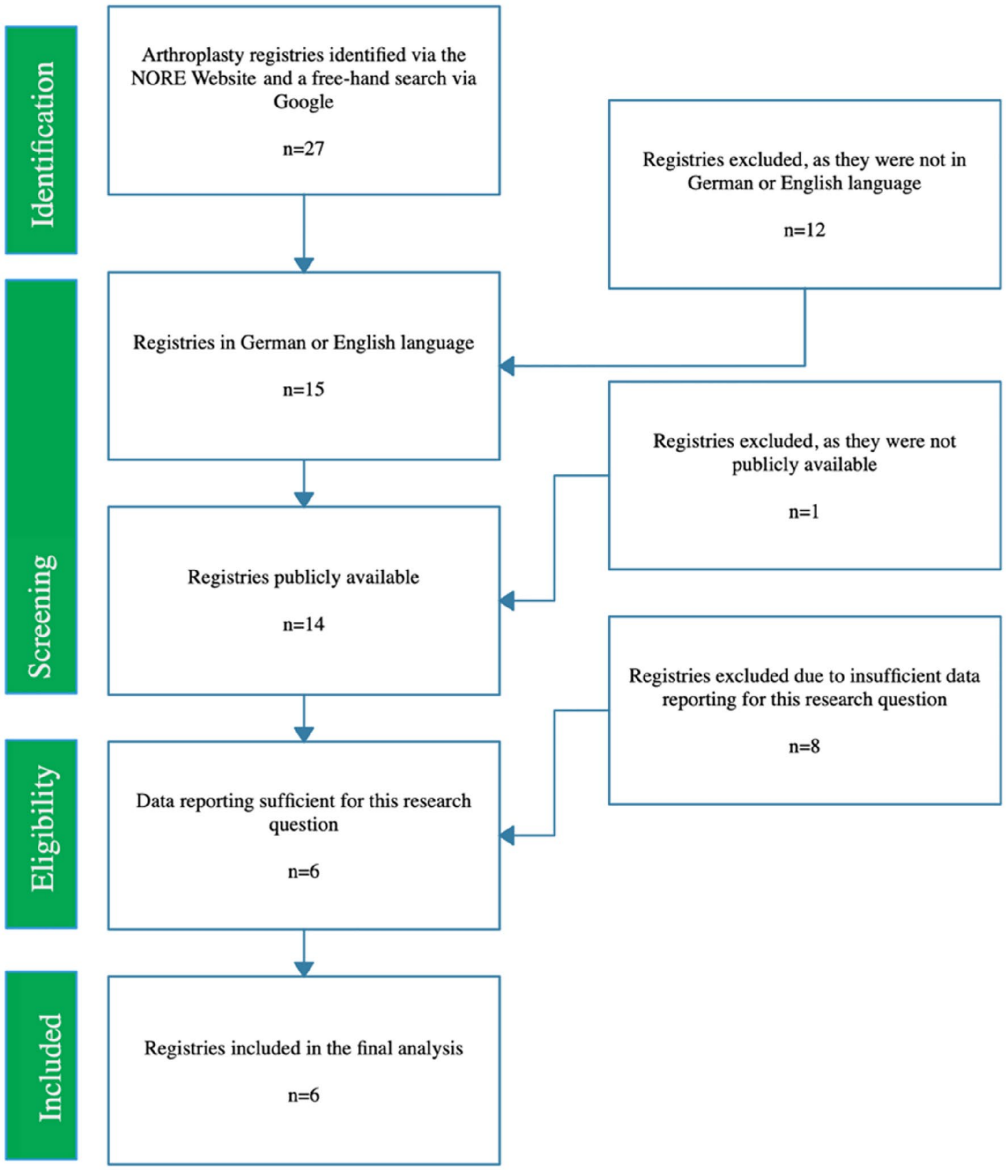
Flow diagram of the identification process of the relevant arthroplasty registers

After initial identification of 27 national or regional knee arthroplasty registers, twelve registers (Belgium, Czech Republic, Denmark, Spain, the Netherlands, France, Hungary, Italy, Slovenia, Valdoltra Slovenia, Pakistan, Saudi Arabia) were excluded as they were not available in German or English language. Subsequently, one registry (Turkey) had to be excluded, as it was not publicly available. Moreover, eight more registers (Finland, Portugal, America, Romania, Scotland, Slovakia, Canada, Sweden) had to be excluded, as data reporting was insufficient regarding this study's research question. Finally, six registers (England and Wales, Australia, Norway, New Zealand, Germany, Switzerland) offered sufficient data according to the inclusion criteria and were thus enclosed in the final analysis. The identification process of the registers that were relevant for this study's research question was depicted in the flow diagram (Fig. 1).

All of the six included registers operate on a national basis.

**Fig. 2:**
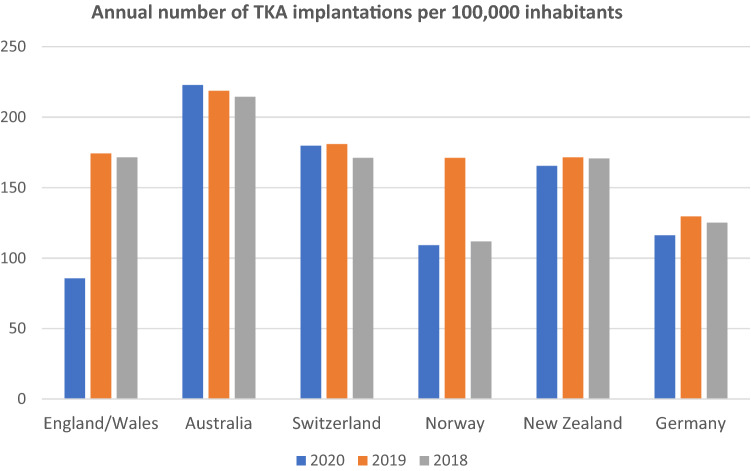
Reported number of annual primary TKA implantations per 100,000 inhabitants in different worldwide arthroplasty registers

A large variation was found concerning the annual number of primary TKA implantations per inhabitant with a range from 112 to 215 per 100,000 in 2018, a range from 130 to 219 per 100,000 in 2019 and a range from 86 to 223 per 100,000 in 2020, as demonstrated in Fig. 2. The lowest number of primary TKA implantations was found in England and Wales in 2020 with 86 per 100,000 inhabitants, while the highest number of TKA implantations was performed in 2020 in Australia, with a frequency of 223 per 100,000.

**Fig. 3:**
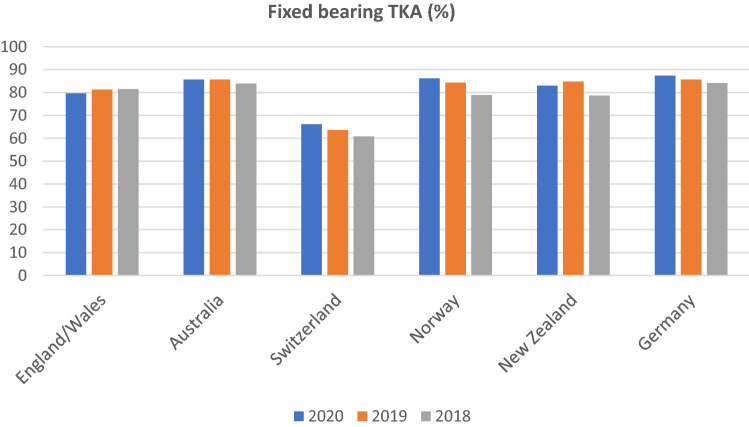
Percentage frequency of fixed-bearing concepts used in primary TKA

**Fig. 4:**
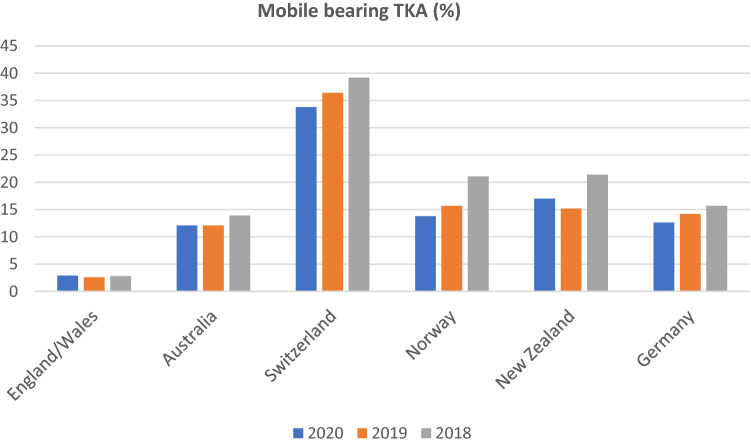
Percentage frequency of mobile-bearing concepts used in primary TKA

In Figs. 3 and 4, the distributions of fixed and mobile-bearing designs used in primary TKA were outlined. In all countries, the dominant type of bearing used for TKA was fixed-bearing design, although the frequency of use decreased slightly over the observation period between 2018 and 2020, except for England and Wales, where it showed a steady increase. Percentage frequencies of fixed-bearing design ranged from 60.8% to 84.1% in 2018, 63.6% to 85.7% in 2019 and 66.2% to 87.4% in 2020. The lowest share between 2018 and 2020 was found in Switzerland, while the highest frequency of use was observed in Germany. A large variation was observed concerning mobile-bearing design, which accounted for 2.8% to 39.2% of bearings in TKA in 2018, 2.6% to 36.4% in 2019 and 2.9% to 33.8% in 2020. It was most often used in Switzerland, while the lowest share was reported in England and Wales.

**Fig. 5:**
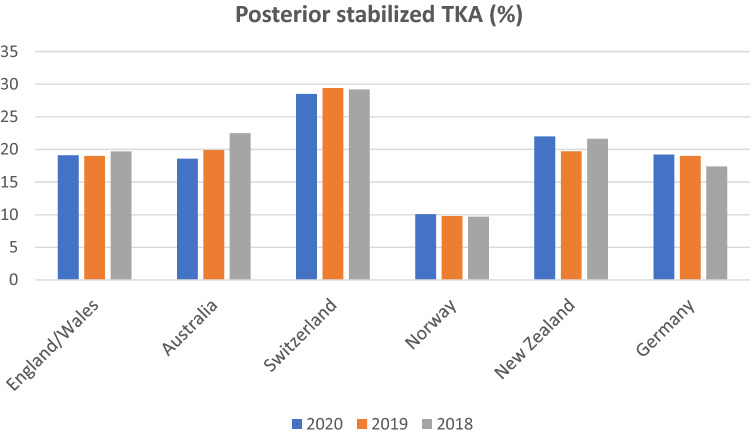
Percentage frequency of posterior-stabilized designs in primary TKA

Some variation was found regarding the use of PS TKA, as its percentage frequency ranged from 9.7% to 29.2% in 2018, 9.8% to 29.4% in 2019 and 10.1% to 28.5% in 2020, as outlined in Fig. 5. The lowest frequency of use between 2018 and 2020 was reported in Norway, while the highest share was found in Switzerland.

Unfortunately, separate information regarding medial pivot design was not given in all registers. However, in those two registers referring to medial pivot design, a share of 9.1% in 2018, 8.6% in 2019 and 8.4% in 2020 was found in Australia, while it only accounted for 1.4% of TKA designs in 2018, 2.1% in 2019 and 2.5% in 2020 in Germany. While the share of medial pivot design in Australia showed a gentle decrease in the observation period, a slight, but steady increase in the use of medial pivot design in Germany was observed.

## Discussion

Worldwide arthroplasty registers are important tools to analyze surgical devices; in this case, different prosthesis designs for TKA and comparative analyses have been used as guidelines in the past [29, 30].

One of the most important findings of this study was that in all included registers, the use of fixed-bearing TKA was the most common with shares ranging from 60.8% to 84.1% in 2018, 63.6% to 85.7% in 2019 and 66.2% to 87.4% in 2020, while mobile-bearing accounted for 2.8% to 39.2% of bearings in TKA in 2018, 2.6% to 36.4% in 2019 and 2.9% to 33.8% in 2020. This indicates that although fixed-bearing was used more often in all analyzed registers, still, a certain variation in the use of mobile-bearing between different countries was detected.

Until now, no significant difference regarding postoperative outcomes between mobile- and fixed-bearing has been reported in studies with high level of evidence [31, 32]. Type of bearing did not seem to influence insert wear and thus loosening of prosthesis, survivorship and clinical and functional outcome [19]. Despite the theoretical advantages of mobile-bearing including reduced polyethylene wear, enhanced contact surface, reduced movement of the femoral component on the surface of the insert and more physiological knee kinematics as well as self-correction of rotational mismatch between tibia and femur, a higher occurrence of bearing dislocation are one of the main concerns associated with it [33–35]. Many bearing dislocations occurred at an early stage after TKA and were attributed to improper surgical technique. Surgical pitfalls likely resulting in this complication comprise mal-rotation of the tibial baseplate and failure to produce properly balanced flexion and extension tension between the femoral- and tibial-bearing interfaces [33, 36]. Especially the higher risk for dislocation in mobile-bearing designs might contribute to why the share of mobile-bearing concepts is quite small throughout all surveyed registers.

In our study, some variation was found regarding the use of PS TKA, as it accounted for 9.7% to 29.2% of TKA in 2018, 9.8% to 29.4% in 2019 and 10.1% to 28.5% in 2020. The lowest share between 2018 and 2020 was reported in Norway, while the highest frequency of use was found in Switzerland.

A meta-analysis of RCTs comparing posterior cruciate-retaining to posterior-stabilized TKA showed that apart from better range of motion and flexion angle for PS design, no significant differences regarding clinical scores, extension angle, complication rate and prosthesis survivorship were found [37]. Studies proposed several explanations, for instance that the joint component gap kinematics has a different pattern in CR and PS TKA and that PS design has more conforming knee kinematics [19, 38]. Moreover, ligament balancing of the posterior cruciate ligament to overcome flexion gap tightness, resulting in poorer flexion angle, is not necessary [39, 40]. Concerning prosthesis survivorship and the need for revision, no significant difference was found in randomized controlled trials (RCTs), so far [37]. An important factor that might contribute to increased revision and prosthesis loosening for PS TKA is possible impingement of the posterior-stabilized peg and the side walls of the inter-condylar housing of the femoral component leading to increased forces at the interface between the tibial polyethylene and the metal tibial tray and hence increased polyethylene wear [41]. Moreover, patients with compromised ligaments might be more suitable for PS design and thus, surgery at a later stage of osteoarthritis might be associated with a higher use of PS design than performing surgery at an earlier stage on younger patients. In contrast, a recent clinical trial by Savov et al. including 248 patients detected higher revision rates for posterior cruciate-retaining than PS TKA for the treatment of valgus osteoarthritis. Regarding clinical outcomes, no difference was found. However, 8.0% of patients in the CR group had to undergo revision surgery due to instability, as compared to no patients in the PS group. The authors concluded that PS TKA might be the more suitable treatment option for valgus cases [42].

In a large survey among Latin American orthopaedic surgeons, the use of different total knee arthroplasty techniques was reported. The survey was completed by 262 surgeons in total. One outstanding finding was that 73% of surgeons used PS design, while 18% used CR and 9% medial pivot design [43]. This is in contrast to the findings of our systematic comparison, where the highest share of PS design used was found in Switzerland, where it only accounted for 28.5% to 29.4% between 2018 and 2020. In all registries included in this analysis, an overwhelming use of CR design was found.

The amounts of prostheses with medial pivot design were only reported in the Australian and German arthroplasty registers, with a share ranging from 8.4% to 9.1% of TKA designs between 2018 and 2020 in Australia, while it only accounted for 1.4% to 2.5% in Germany during the same time period. Medial pivot design is relatively new compared to the other mentioned concepts and studies presenting especially long-term outcomes are still sparse. Hence, also the specification of medial pivot design in arthroplasty registers was still rare to be found. These implants are designed to specifically reproduce more natural physiological kinematics of the knee joint, as it results in the anatomical anterior sliding movement and lacks the paradoxical rollback of the femoral condyles in conventional TKA [44].

The aim of the medial pivot concept was to replicate the anatomical tibial plateau design by including an insert with a deep medial compartment highly congruent and a lateral compartment with less conformity [45]. Knee motion results in a “ball-in-socket articulation” in the medial compartment and rolling from anterior to posterior in the lateral compartment, mimicking normal knee kinematics [46]. Theoretical advantages of medial pivot design include restoration of normal knee kinematics and stability, optimization of range of motion, reduced wear and preservation of bone stock for primary and revision TKAs [47–50]. One recent study with longer-term results by Macheras et al. with a minimum follow-up period of 15 years reported excellent pain relief in 93% of patients and excellent recovery of function in 94% of patients [51]. Moreover, objective as well as subjective clinical outcome scores were significantly improved and ROM increased from 85° to 120° on average. Similar results were demonstrated by Karachalios et al. with a follow-up period of 11–15 years [52]. Another recent systematic review by Alessio-Mazzola et al. elaborated the clinical and radiological outcomes as well as survivorship of medial pivot design TKA. Their most important findings included excellent prosthesis survivorship and low revision rates of 1.9% after 10 years. Moreover, clinical and radiological results were good, indicating many potential benefits associated with medial pivot design, which still need to be confirmed in larger trials, however [53]. What remains controversial is the fact if the PCL should be retained or sacrificed with this prosthesis, with the current opinion in favor of substitution of the PCL. Two recent randomized controlled trials (RCT) have compared medial pivot against PS design. Kulshrestha et al. have analyzed 40 patients in their RCT and found that patients after medial pivot design, TKA had similar patient-reported outcomes as those with PS design TKA. Additionally, they had better results regarding getting up from a chair in the timed up-and-go test and concerning walking speed in the self-paced walk test. However, the gain in knee flexion compared to baseline was significantly greater in the PS group. [54] On the other hand, another recent RCT by Chang et al. showed that patients undergoing medial pivot TKA had comparable range of motion (ROM) at one year and two years after surgery. Moreover, patient-reported outcome measures, postoperative limb alignment or complications offered no statistically significant differences. [55] Therefore, as these two RCTs yielded quite heterogeneous results, future studies with larger patient collectives will be necessary to confirm the significance of these findings from previous RCTs.

Consequently, the most important factor to determine the optimal TKA design is long prosthesis survival, along with solid function. Factors influencing prosthesis survival include implant design, patient selection and surgical technique. From the current point of view, an individual approach taking all these mentioned factors into account is probably needed to choose what’s best for the individual patient to make durability and functionality of TKA as long as possible.

There were several limitations to this present study. First, the quality of this study depends on the quality of the primary register data included. It is unclear if the numbers reported in the registers truly reflect the exact number of surgeries performed in every country, respectively. Second, due to our inclusion criteria, only six registers were included altogether, as some only listed the brands of the used implants, but for a certain brand, different designs can be used, for example mobile-bearing as well as fixed-bearing. This is the reason why, for example, the Swedish register could not be included. Furthermore, the reporting schemes of different design concepts were not standardized between different countries. Third, especially for medial pivot design, numbers are quite small as this concept is relatively new and several registers have not differentiated between medial pivot and other designs. Additionally, registry data are partially incomplete; thus, the term "bearing type unknown" is presented in Fig. 3. Moreover, in addition to not reporting all surgeries performed in the respective country, registries also do not state how many hospitals are not included.

In conclusion, the comparison of arthroplasty registers from England and Wales, Australia, Norway, New Zealand, Germany and Switzerland revealed large differences regarding the application of posterior-stabilized designs, but also common ground considering the overwhelming use of fixed-bearing inserts, which, when inserted correctly, eradicate the potential complication of bearing dislocation. Arthroplasty registers offer a real-world clinical perspective with the aim to improve quality and patient safety.

## Data Availability

Data and materials are stored by the first author electronically.
